# Expression and gene regulatory network of S100A16 protein in cervical cancer cells based on data mining

**DOI:** 10.1186/s12885-023-11574-y

**Published:** 2023-11-17

**Authors:** Haibin Zhang, Yongxiu Yang, Wenhu Xing, Yufeng Li, Shan Zhang

**Affiliations:** 1 The First School of Clinical Medicine of Lanzhou University, Lanzhou, 730013 Gansu Province China; 2 Department of Gynecology, the Second Hospital of Lanzhou University, Lanzhou, 730013 Gansu Province China; 3 Department of Obstetrics and Gynecology, The First Hospital of Lanzhou University, Lanzhou, 730013 Gansu Province China; 4 The Key Laboratory of Gynecological Tumors in Gansu Province, Lanzhou, 730013 Gansu Province China; 5 The Second School of Clinical Medicine of Lanzhou University, Lanzhou, 730013 Gansu Province China

**Keywords:** Cervical cancer, S100A16 protein, Molecular mechanisms, TCGA

## Abstract

S100A16 protein belongs to the S100 family of calcium-binding proteins, which is widely distributed in human tissues and highly conserved. S100 calcium-binding proteins possess broad biological functions, such as cancer cell proliferation, apoptosis, tumor metastasis, and inflammation (Nat Rev Cancer 15:96–109, 2015). The S100A16 protein was initially isolated from a cell line derived from astrocytoma. The S100A16 protein, consisting of 103 amino acids, is a small acidic protein with a molecular weight of 11,801.4 Da and an isoelectric point (pI) of 6.28 (Biochem Biophys Res Commun 313:237–244, 2004). This protein exhibits high conservation among mammals and is widely expressed in various human tissues (Biochem Biophys Res Commun 322:1111–1122, 2004). Like other S100 proteins, S100A16 contains two EF-hand motifs that form a helix-loop-helix structural domain. The N-terminal domain and the C-terminal domain of S100A16 are connected by a "hinge" linker.S100A16 protein exhibits distinct characteristics that distinguish it from other S100 proteins. A notable feature is the presence of a single functional Ca2 + binding site located in the C-terminal EF-hand, consisting of 12 amino acids per protein monomer (J Biol Chem 281:38905–38917, 2006). In contrast, the N-terminal EF-hand of S100A16 comprises 15 amino acids instead of the typical 14, and it lacks the conserved glutamate residue at the final position. This unique attribute may contribute to the impaired Ca2 + binding capability in the N-terminal region (J Biol Chem 281:38905–38917, 2006). Studies have shown an integral role of S100 calcium-binding proteins in the diagnosis, treatment, and prognosis of certain diseases (Cancers 12:2037, 2020). Abnormal expression of S100A16 protein is implicated in the progression of breast and prostate cancer, but an inhibitor of oral cancer and acute lymphoblastic leukemia tumor cell proliferation (BMC Cancer 15:53, 2015; BMC Cancer 15:631, 2015). Tu et al. (Front Cell Dev Biol 9:645641, 2021) indicate that the overexpression of S100A16 mRNA in cervical cancer(CC) such as cervical squamous cell carcinoma and endocervical adenocarcinoma as compared to the control specimens. Tomiyama N. and co-workers (Oncol Lett 15:9929–9933, 2018) (Tomiyama, N) investigated the role of S100A16 in cancer stem cells using Yumoto cells (a CC cell line),The authors found upregulation of S100A16 in Yumoto cells following sphere formation as compared to monolayer culture.Despite a certain degree of understanding, the exact biological function of S100A16 in CC is still unclear. This article explores the role of S100A16 in CC through a bioinformatics analysis. Referencing the mRNA expression and SNP data of cervical cancer available through The Cancer Genome Atlas (TCGA) database, we analyzed S100A16 and its associated regulatory gene expression network in cervical cancer. We further screened genes co-expressed with S100A16 to hypothesize their function and relationship to the S100A16 cervical cancer phenotype.

Our results showed that data mining can effectively elucidate the expression and gene regulatory network of S100A16 in cervical cancer, laying the foundation for further investigations into S100A16 cervical tumorigenesis.

## Introduction

Cervical cancer is one of the most common malignant tumors in gynecology and a serious threat to women’s health worldwide; it ranks fourth among cancers in women and is especially prevalent in the developing world [1]. Improvement in cervical cancer screening and early detection methods and the availability and increased administration of the HPV vaccine in recent years, has reduced the cervical cancer burden on public health, but its pathogenic mechanisms remain unclear. The main treatment modalities for cervical cancer include surgery, chemotherapy, radiation therapy, and targeted therapy. Clinical research on the etiology of cervical cancer is still ongoing.

Recent advancements in gene microarray, high-throughput sequencing techniques, and bioinformatics continue to provide novel approaches for disease research [1]. Our study screened the differential gene expressions in cervical cancer and healthy tissues based on the TCGA database. Through analysis of the copy number variation of hub genes, gene set variations, immune cell infiltration, weighted gene co-expression network analysis (WGCNA), and gene module function enrichment, we explored the related molecular mechanisms and provided a new research approach to the diagnosis and treatment of cervical cancer.

## Materials

### TCGA data acquisition and differential analysis

The TCGA database (https://portal.gdc.cancer.gov/.) is the largest cancer gene information resource for information pertaining to gene expression data, copy number variation, and single nucleotide polymorphisms (SNP). We downloaded the original mRNA expression data and known SNPs in cervical cancer for subsequent analysis. A total of 309 specimens were collected (normal group, *n* = 3; tumor group, *n* = 306) to analyze the expression differences of S100A16. We downloaded the series matrix files of GSE44001 from the NCBI GEO public database. The annotation platform was GPL14951. The data for 300 cervical cancer patients complete with expression profiles and survival information were retrieved. This study was approved by the Ethics Committee.

### Expression of S100A16 protein in cervical cancer tissues

A total of 63 pairs of pathological slides were collected from cervical cancer surgical patients at the Second Hospital of Lanzhou University from January 2020 to July 2022. The pathology confirmed the presence of cervical cancer. A microscopic imaging system was used to capture images of the slides. Initially, the entire tissue was observed at 100 × magnification, followed by image acquisition at 400 × magnification. The results were photographed and recorded. After completing immunohistochemical staining on tissue microarrays (primary antibody at a ratio of 200:1), the cell nuclei were stained blue with hematoxylin, and positive expression shown by DAB staining appeared as brownish-yellow. Each specimen was photographed at 200 × magnification. The staining results were blindly assigned by three pathologists with independent diagnostic qualifications. The scoring criteria included: (1) staining intensity of the protein, and (2) percentage of positive cells. The product of (1) and (2) was used to determine the score, which was then averaged and grouped. The presence of a yellow or brown area in S100A16 protein immunohistochemical staining was considered positive. The scoring criteria for protein staining intensity were as follows: no staining (0 points), light staining (1 point), moderate staining (2 points), and strong staining (3 points). The scoring criteria for the percentage of positive cells were as follows: > 10% (1 point), 11%-50% (2 points), 51%-75% (3 points), and > 75% (4 points). Additionally, in order to understand the relationship between the expression level of S100A16 protein and clinical indicators, the expression of S100A16 protein was divided into a high-expression group (IHC score ≥ 12) and a low-expression group (IHC score < 12).

### Co-expression analysis

The co-expression status of S100A16 in cervical cancer was analyzed with a pre-established filter and selection threshold of a 0.3 correlation coefficient and *p*-value of 0.05. After selecting the most significant genes with S100A16 expression, the “corrplot” and “circlize” packages were used to develop the heatmap and circular plot for S100A16 correlative analysis.

### Immune cell infiltration analysis

The CIBERSORT algorithm was used to analyze the RNA-seq data of cervical cancer patients and identify relative proportions of 22 immune cells to uncover any correlation in gene expression level and immune response.

### Gene set variation analysis (GSVA)

GSVA is a non-parametric unsupervised method to assess the enrichment of transcriptomic gene sets. Through comprehensively scoring the gene sets of interest, GSVA converts gene-level changes to pathway-level changes and then determines the biological function of the sample. We downloaded gene sets from the Molecular Signatures Database (MSigDB), developed a comprehensive score for each gene set using the GSVA algorithm, and evaluated the potential biological function changes across different samples.

### Gene set enrichment analysis (GSEA)

GSEA is commonly used for disease classification and is closely related to biological significance. In this study, patients were divided into high and low S100A16 expression groups. The differences in the signaling pathways between the high and low expression groups were further analyzed using GSEA. The background gene set and pathway subtypes were the version 7.0 annotated gene sets downloaded from the MSigDB database. Differential expression analysis of signaling pathways between subtypes was performed, and the significantly enriched gene sets (adjusted *p* value < 0.05) were sorted based on consensus scoring.

### Drug sensitivity analysis

Based on the largest pharmacogenomics database (https://www.cancerrxgene.org/) we used the R software package “pRRophetic” to predict the chemotherapy sensitivity for each tumor specimen. The estimated IC50 with each specific chemotherapy drug was obtained using the regression method, and 10-fold cross-validation was performed to test the regression and prediction accuracy using the GDSC training sets. All parameters had the default and “combat” values selected removing the batch effect and taking the average of duplicate gene expression.

### Nomogram model construction

A nomogram was built based on regression analysis of gene expression level and clinical symptoms. Line segments were used with tick marks to draw on the same plane according to a predetermined scale, thereby conveying the interrelationships between variables in a predictive model. The multivariate regression model assigned scores to individual value levels of each influencing factor based on its degree of contribution in the model to the outcome variable (i.e., magnitude of the regression coefficient). The individual scores were added together to obtain the total score, ultimately allowing for calculation of the predicted value.

### WGCNA

By constructing a weighted gene co-expression network, we investigated S100A16 and any associated genes. The co-expression networks for all genes in the cervical cancer datasets were constructed using the WGCNA-R package, and the top 10,000 genes with variance were screened using this algorithm with a soft threshold set to 5. The weighted adjacency matrix was converted to a topological overlap matrix (TOM) that estimated network connectivity, and hierarchical clustering constructed a tree of the TOM matrix. Different branches of the clustering tree represented different gene modules that were further differentiated by color. Based on their weighted correlation coefficients, the genes were classified by expression patterns and combined with similarly expressing genes into one module, grouping all genes into multiple modules.

### Functional enrichment analysis of gene modules

R package “ClusterProfiler” was used to annotate the biological function of the modular genes selected from the WGCNA key (the magenta module was most correlated with S100A16). Gene Ontology (GO) and Kyoto Encyclopedia of Genes and Genomes (KEGG) was used to evaluate the associated functional categories. The GO and KEGG enriched pathways with both p and Q values below 0.05 were considered significant categories.

### Statistical analysis

Statistical analysis was performed using R programming language (version 4.2.1). For immunohistochemical scores, non-parametric test for paired data (Wilcoxon signed rank test) was performed using SPSS (version 25.0). *P* < 0.05 was considered statistically significant.

## Results

### The expression pattern and prognosis of S100A16 in cervical cancer

**Fig. 1:**
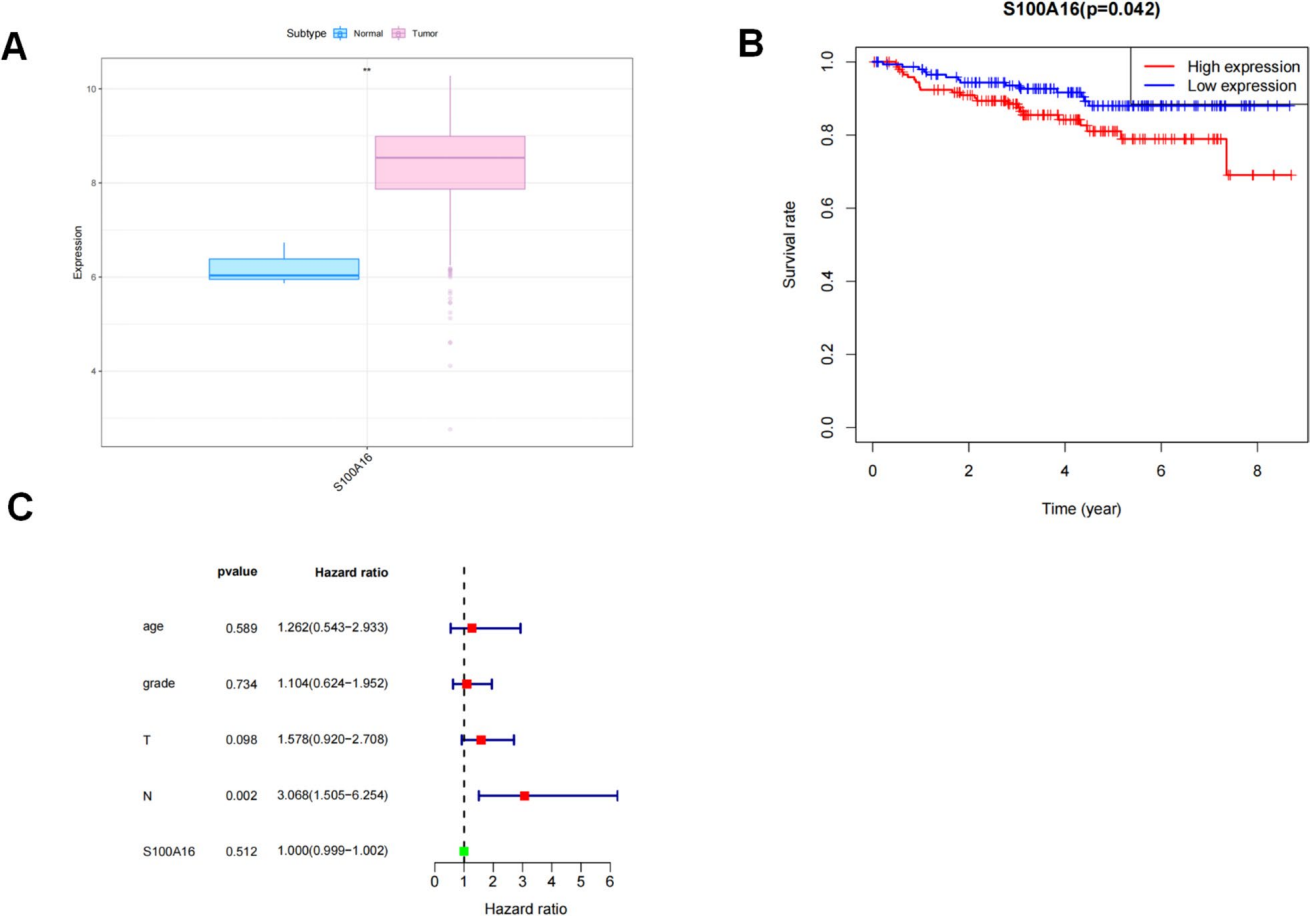
**A** Expression of S100A16 mRNA in tumor and normal tissues. Blue represents control tissues, and red represents tumor tissues. **B** Increased S100A16 expression in cervical cancer is associated with a poor prognosis. **C** Univariate and multivariate forest plots. Green indicates protective factors, and red indicates risk factors

S100A16 expression was significantly upregulated in cervical cancer specimens (Fig. 1A). Our survival analysis, ranked by degree of S100A16 gene expression, indicated that in the GSE44001 dataset, the overall survival (OS) within the high S100A16 expression group was significantly shorter than groups with low S100A16 expression (Fig. 1B). Using clinical information and S100A16 gene expression level, we established univariate and multivariate Cox regression models and constructed forest plots that showed N in cervical cancer patients was associated with the risk and had a statistical difference between the groups (Fig. 1C).

### Immunohistochemical analysis of S100A16 in cervical cancer

**Fig. 2:**
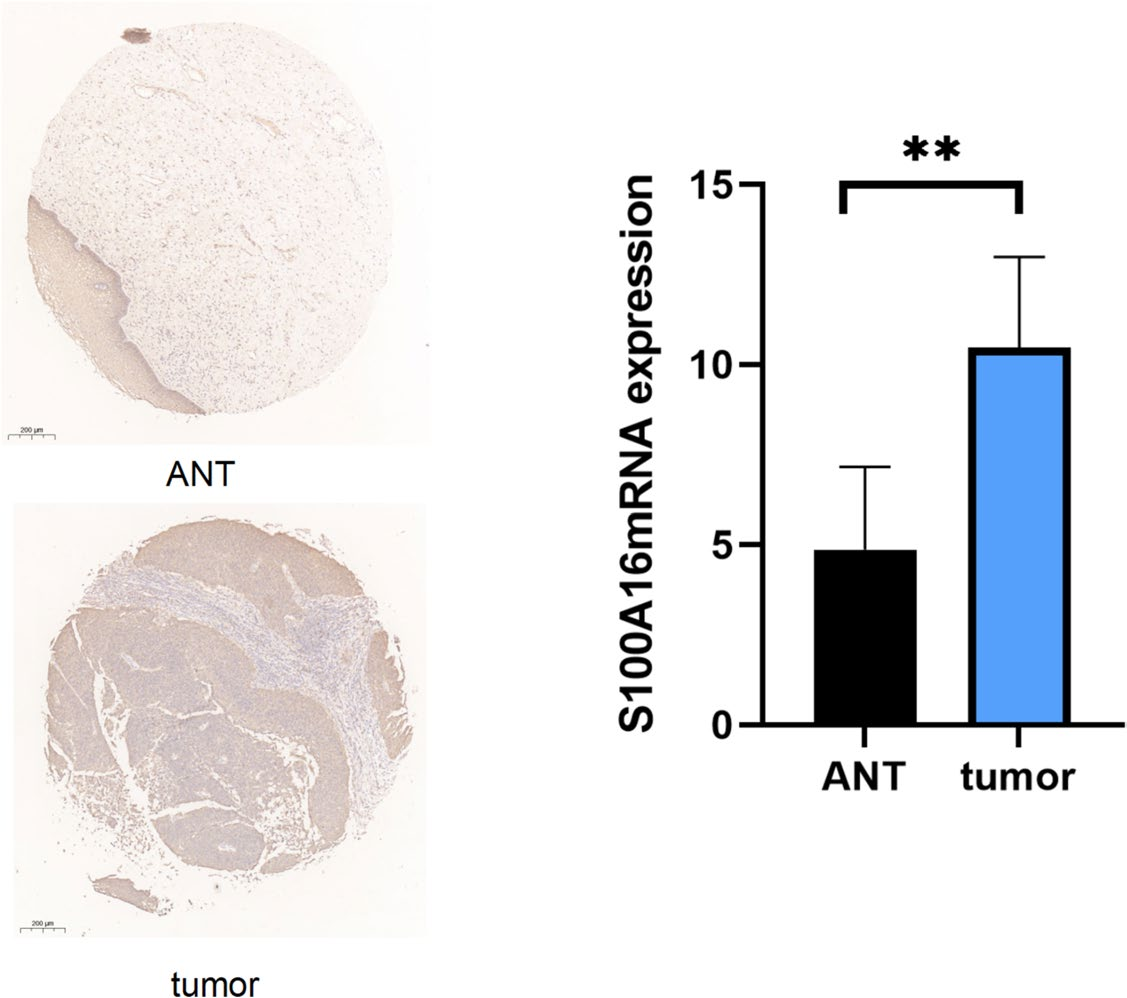
Expression level of S100A16 protein in human cervical cancer tumor specimens (tumor) and corresponding adjacent normal tissue specimens (ANT) detected by immunohistochemical staining

Immunohistochemical staining of cervical cancer cells and the corresponding adjacent normal healthy cervical tissue specimens (ANT) was performed to investigate whether the S100A16 protein expression was altered in the tissues of cervical cancer patients. Our research revealed a statistically significant (*p* < 0.05) increase in the levels of S100A16 protein in 63 tumor specimens compared to the corresponding cervical paracancerous specimens (Fig. 2). According to the scoring criteria of immunohistochemistry, the 63 cervical cancer patients were divided into two groups: a high-expression group with 43 cases (68%) and a low-expression group with 20 cases (32%). The results indicated a significant correlation between the expression level of S100A16 protein and the pathological grading and depth of muscular infiltration in the cervix (*P* < 0.05). However, there was no significant difference in age, lymph node metastasis, tumor size, and tissue type among the patients (*P* > 0.05) (Table 1).

**Table 1:** The relationship between the expression of S100A16 and the clinical pathological indicators in cervical cancer patients

clinical parameters	S100A16 expression		c2	*P*-value
	LOW (*n* = 20)	High (*n* = 43)		
age				
≤ 50 years	10	23	0.067	0.796
> 50 years	10	20		
pathology grading				
G1	1	1	20.989	0.000
G2	15	30		
G3	4	12		
Lymph node metastasis				
yes	2	6	0.107	0.744
no	11	44		
Involved with vessel				
yes	4	23	0.977	0.323
no	9	27		
Invasion depth				
≥ 1/2	4	25	5.751	0.016
< 1/2	14	20		
Tumor in diameter				
≥ 4 cm	3	14	0.456	0.5
< 4 cm	10	36		
Issue types				
squamous carcinoma	12	47	1.576	0.665
Adeno squamouses carcinoma	0	1		
endometrioid adenocarcinoma	0	1		
adenocarcinoma	1	1		

### Co-expression gene analysis of S100A16 in cervical cancer

**Fig. 3:**
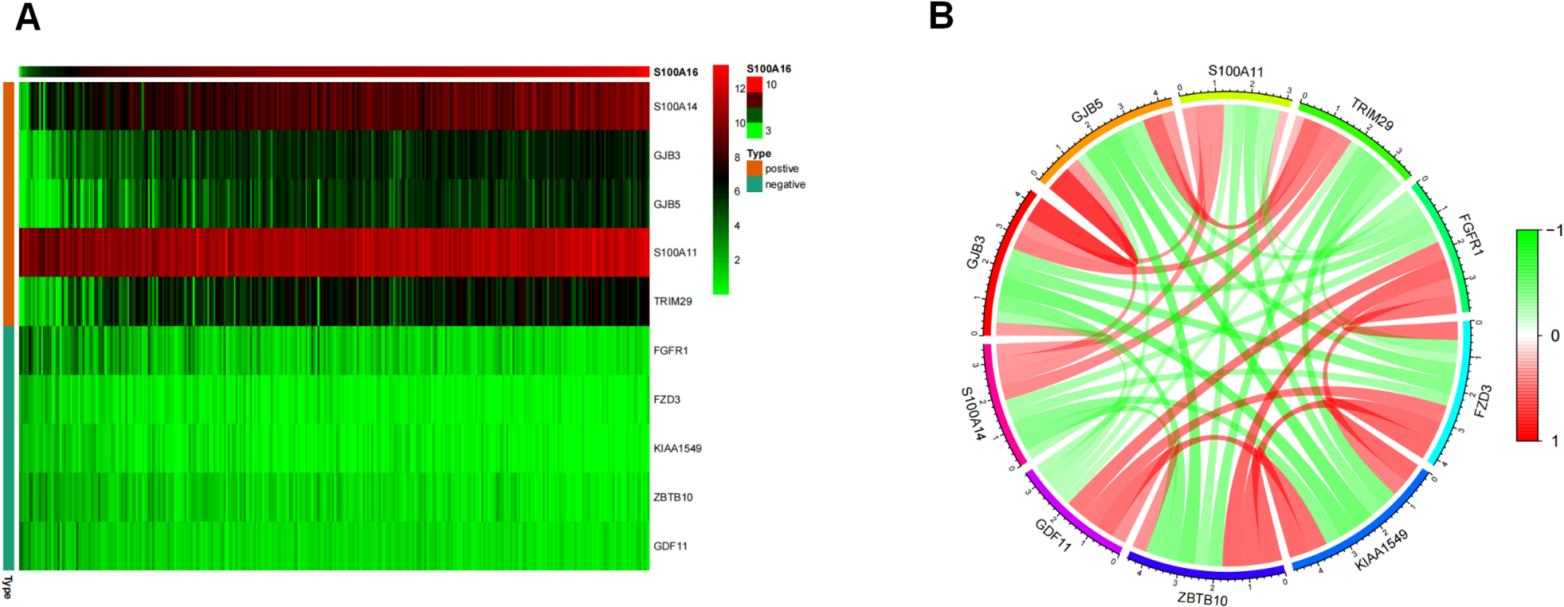
**A** Gene heat map. The heatmap shows the top 10 genes with the most significant correlation in CESC patients with high or low expression of S100A16. **B** Circular plot showing the network of important S100A16-related genes in the high or low expression group of S100A16 in CESC patients

We further explored the co-expression network of S100A16 using the expression profiles of cervical cancer patients annotated in the TCGA database. A total of 2,934 genes that were significantly correlated (correlation coefficient filter criterion of 0.3 and *p* value of 0.05) with S100A16 expression were screened and resultant heatmaps of the top 5 genes with positive or negative correlation coefficients (Fig. 3A) along with co-expression correlative circular charts are shown in Fig. 3B.

### The relationship between S100A16 and immune cell infiltration in cervical cancer

**Fig. 4:**
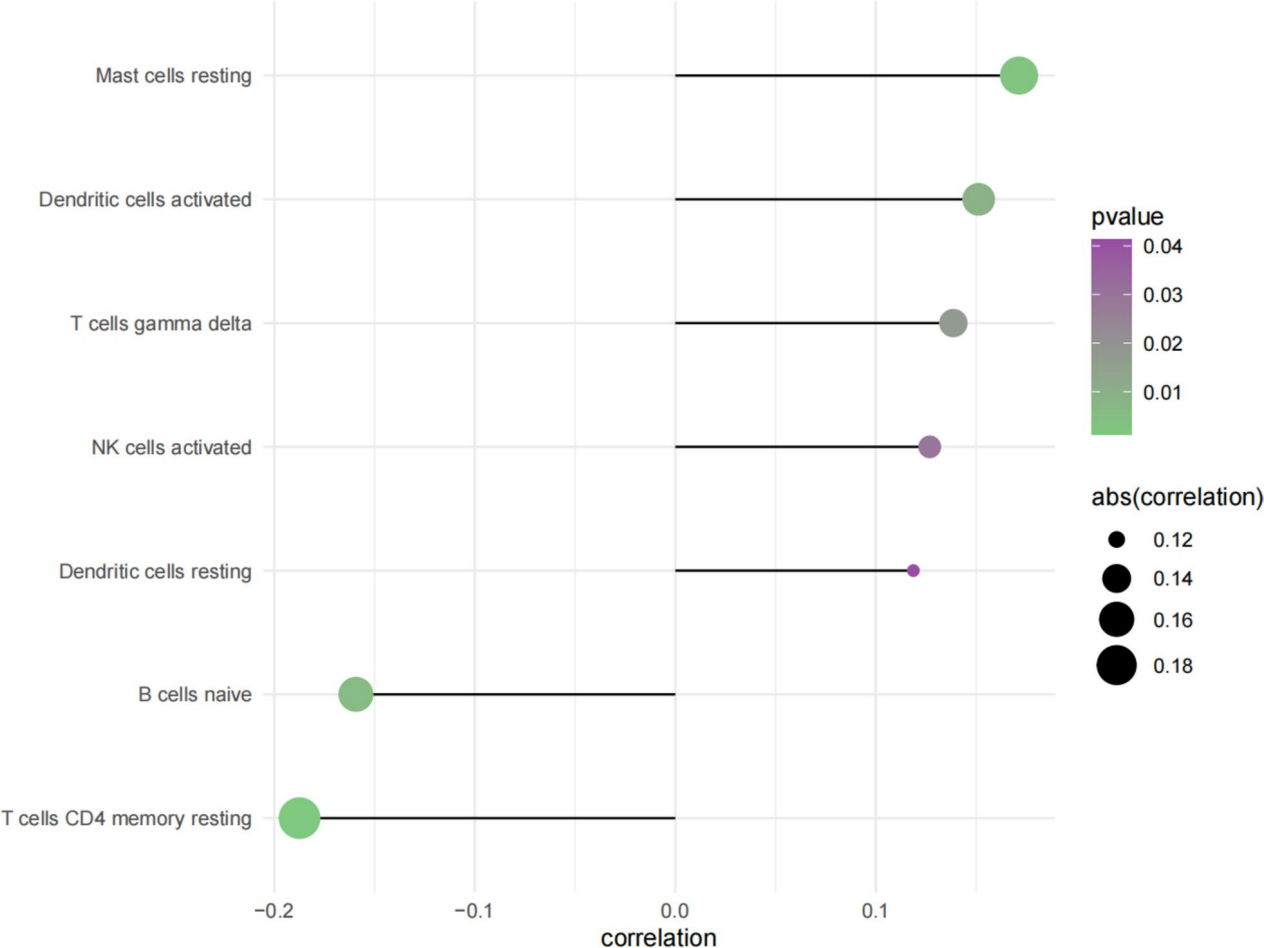
Correlation between S100A16 gene expression and immune cell content

The tumor microenvironment consists primarily of cancer cells and tumor-associated fibroblasts, immune cells, extracellular matrix, growth factors, and inflammatory factors. The composition and unique physiochemical properties of the tumor microenvironment significantly affects cancer diagnosis, survival outcome, and clinical sensitivity to treatment. By analyzing the relationship between S100A16 and the subsequent immune response within the TCGA datasets we probed the potential cancer progression molecular mechanisms influenced by S100A16. Specifically, S100A16 had significant positive correlations to population of resting mast cells and activated dendritic cells and had significant negative correlations with naïve B cells and resting CD4 memory T cells (Fig. 4).

### The signaling pathways related to S100A16 in cervical cancer

**Fig. 5:**
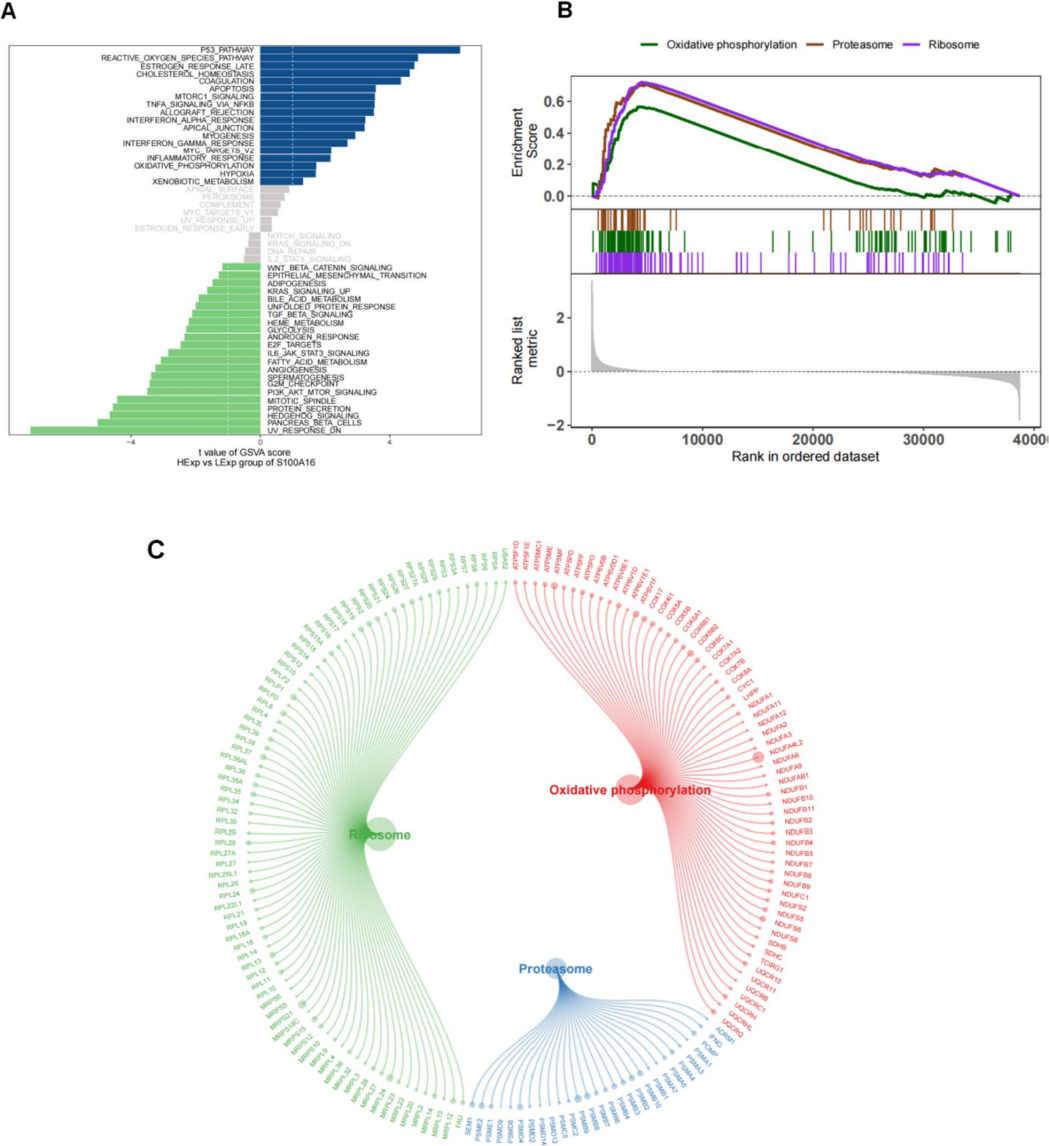
**A** Important biological pathways related to S100A16 were obtained by GSVA in CESC; **B** S100A16 expression was positively correlated with the oxidative phosphorylation, proteasome, and ribosome signaling pathways. **C** Correlation of the genes of the oxidative phosphorylation, proteasome, and ribosome

We examined the specific signaling pathways associated with S100A16 to explore the underlying molecular mechanisms by which S100A16 influenced tumor progression. The GSVA results showed that patients with high S100A16 expression had enrichment in several signaling pathways, such as the p53 regulatory pathway and increased levels of reactive oxygen species (Fig. 5A). Moreover, GSEA indicated S100A16 enrichment in such signaling pathways as oxidative phosphorylation, proteasomes, and ribosomes (Fig. 5B, C). These findings suggest S100A16 involvement in cervical cancer development through such pathways.

### Mutation analysis

**Fig. 6:**
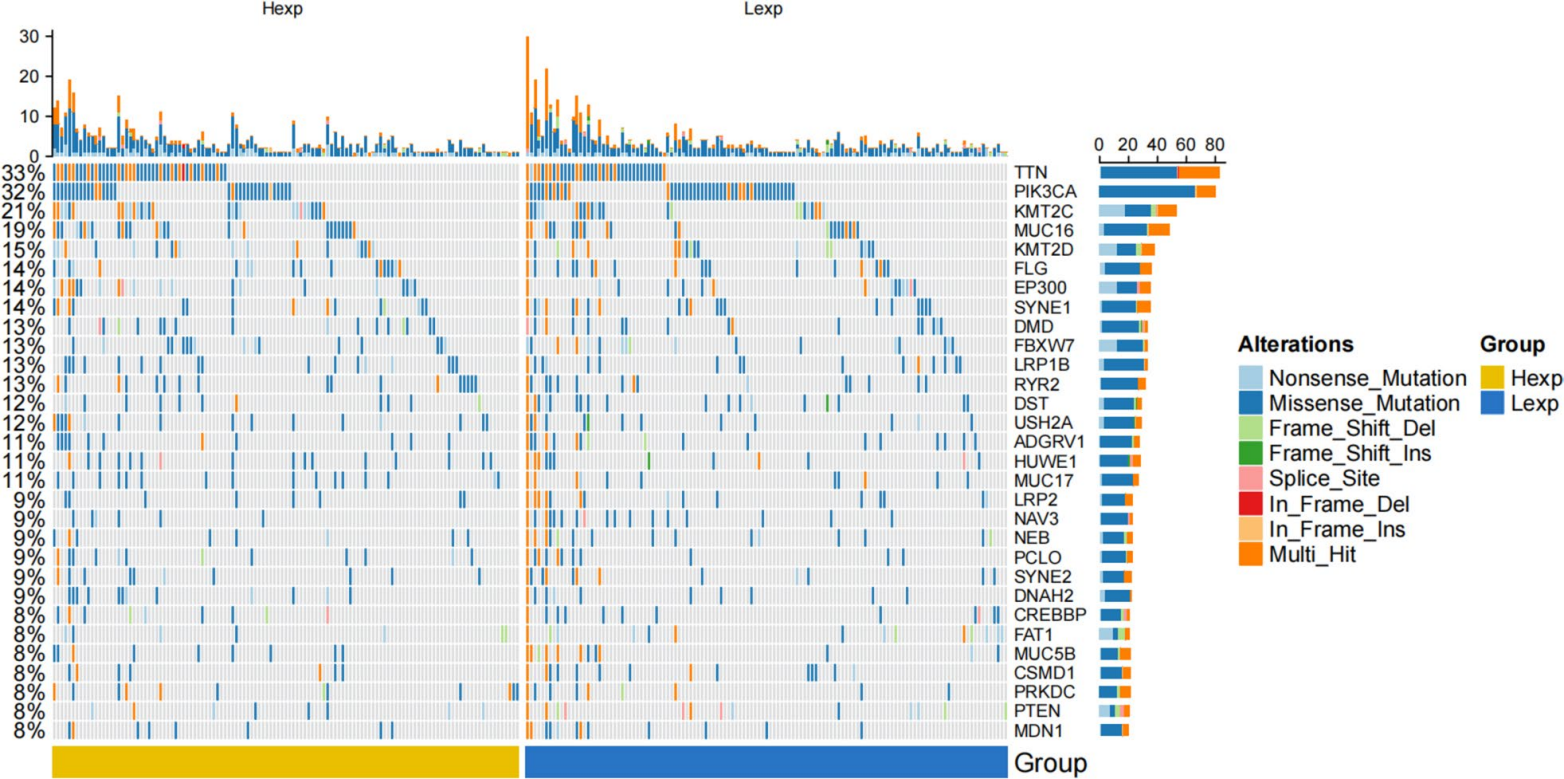
Waterfall plot of S100A16-related mutation genes obtained through mutation analysis in CESC

We downloaded the processed SNP-related data of cervical cancer, selected the top 30 genes with relatively high mutation frequency, compared the differences of the mutated genes between the two groups, and constructed the mutational landscape map using the R package ComplexHeatmap (Fig. 6). Our results showed differences in gene mutation frequency between high and low S100A16 expression levels.

### The relationship between chemotherapy drugs and S100A16 expression in cervical cancer

**Fig. 7:**
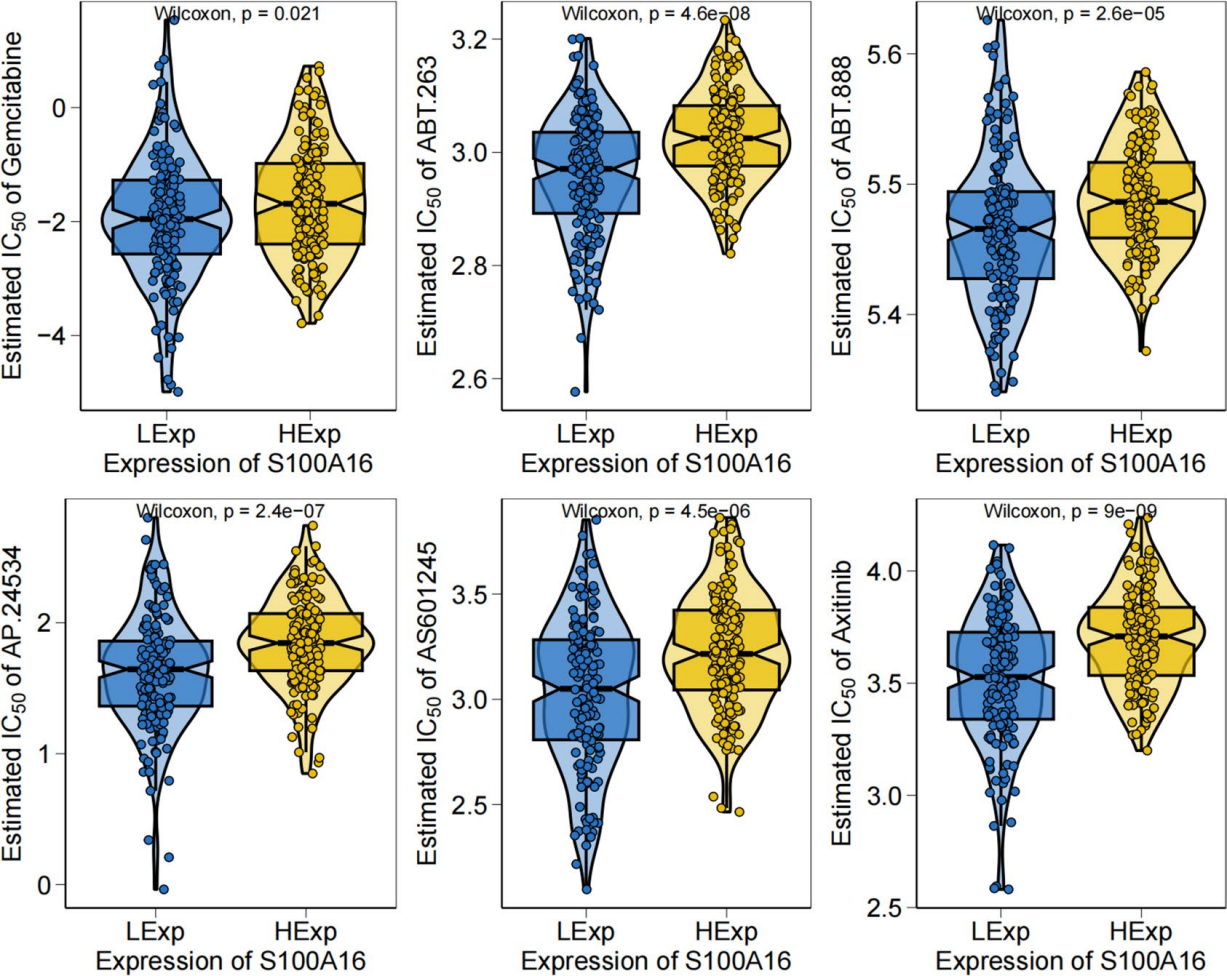
Chemotherapy drug sensitivity analysis based on S100A16 expression

The therapeutic benefit of surgery combined with chemotherapy in early-stage cervical cancer is clear. Based on drug sensitivity data in the GDSC database, our study predicted the chemotherapy sensitivity of each tumor specimen using the R package pRRophetic to further investigate any relation between S100A16 expression levels and sensitivity to common chemotherapeutic drugs. Our results showed a relation in S100A16 and sensitivity to gemcitabine, ABT.263, ABT.888, AP.24534, AS601245, and axitinib (Fig. 7).

### Nomogram model for cervical cancer patients

**Fig. 8:**
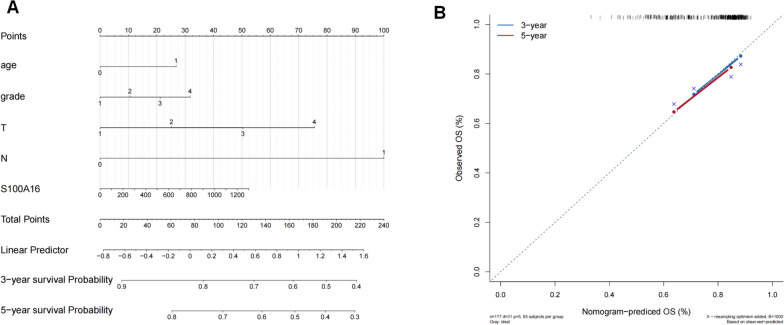
**A** Nomogram model for predicting 3-year and 5-year survival rates of CESC patients (**B**) Calibration curve for 3-year OS based on the nomogram model

Based on the S100A16 expression levels, we presented the results of our regression analysis in the form of a nomogram. Regression analysis of patients in our study showed that the values of the different clinical indicators of cervical cancer and the distribution of S100A16 expression had varying degrees of contribution throughout the scoring process (Fig. 8A). Predictive analysis for 3-year and 5-year OS (Fig. 8B) rates indicated a trend in our nomogram model-predicted OS that was similar to the actual observed OS.

### Weighted gene co-expression network analysis and functional enrichment analysis

**Fig. 9:**
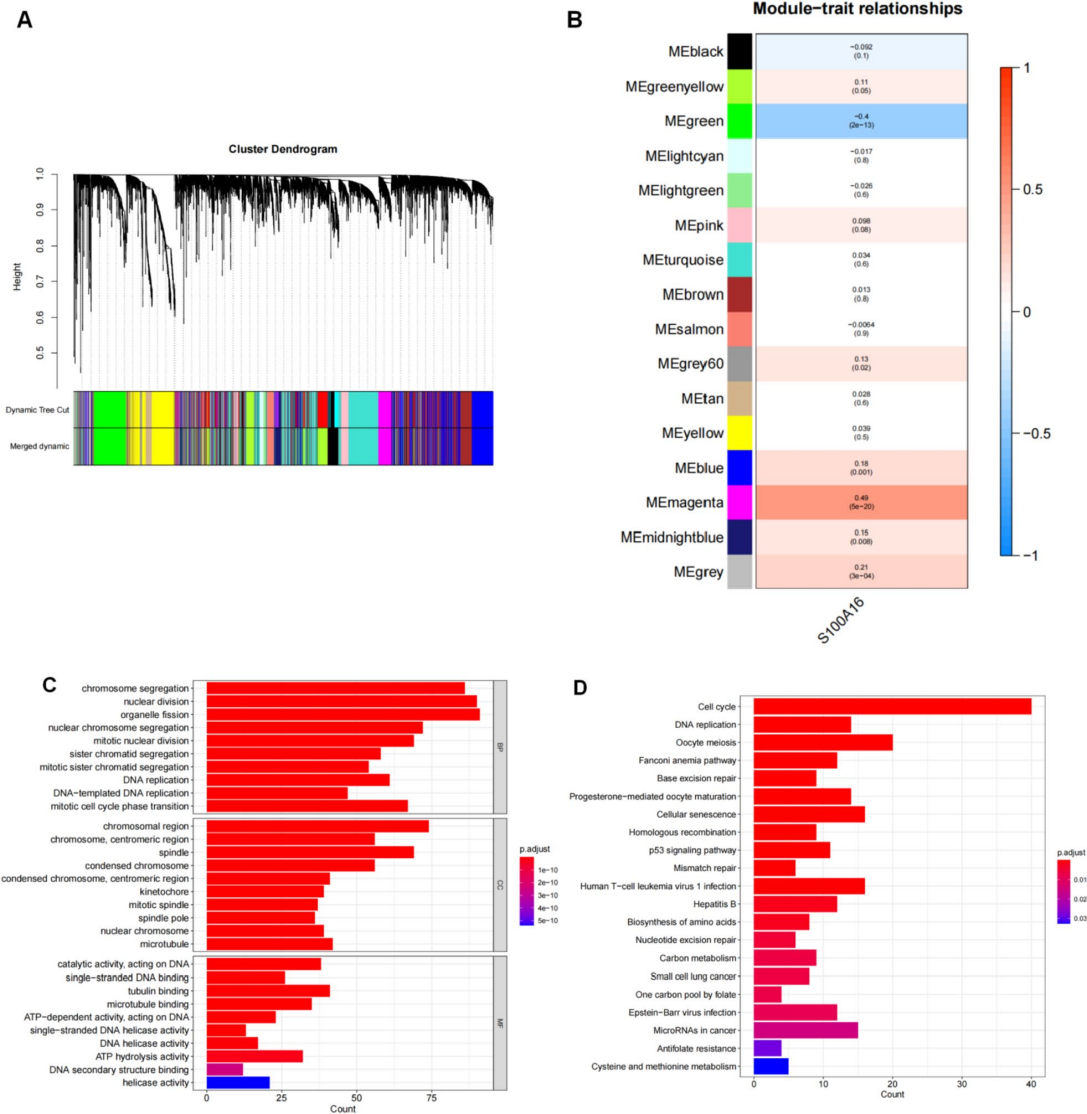
**A** Dendrogram of sample clustering in CESC; **B** Gene set enrichment analysis (GSEA) of S100A16-related biological pathways in CESC; **C** GO analysis results of differentially expressed genes; **D** KEGG analysis results of differentially expressed genes

To determine the co-expression network of S100A16, we performed WGCNA. The soft threshold β was determined by the function “sft$powerEstimate,” with the soft threshold set to 5. Subsequently, the gene modules were inspected based on the TOM matrix. During analysis, a total of 16 gene modules were detected, and we found that the magenta module had the highest correlation with S100A16 (Fig. 9A, B). Subsequent pathway analysis using magenta modular genes was performed, and the GO results showed enriched genes primarily in pathways such as chromosome segregation, nuclear division, and organelle fission (Fig. 9C). Additionally, KEGG results showed enrichment mainly in pathways such as cell cycle, DNA replication, and oocyte meiosis (Fig. 9D).

## Discussion

Cervical cancer is one of the most common malignant tumors in women. At present, persistent HPV infection is the most important risk factor leading to cervical cancer. However, other factors such as age and genetic factors also influence the progression of cervical cancer [2, 3]. Current standard of care for cervical cancer is surgery and chemotherapy; however, the prognosis of cervical cancer patients remains uncertain. Therefore, it is particularly imperative to search for new diagnostic methods, markers of tumor progression, and novel therapeutic targets.

As a member of S100 proteins, S100A16 was initially isolated from astrocytomas [4, 5]. It can promote chromosomal rearrangement and instability and in this respect, it is oncogenic [6, 7]. In addition, S100A16 showed aberrant expressions in different neoplastic tissues further suggesting its involvement in tumorigenesis [8–15]. Previous studies have found a correlation between S100A14 and the occurrence and progression of cervical cancer. Overexpression of S100A14 is closely associated with the staging and lymph node metastasis of cervical cancer. S100A14 has been shown to promote cell cycle progression, cell growth, migration, and invasion of cervical cancer cells [16]. In this study, a close correlation was observed between the high expression of S100A16 and GJB3. GRAEBER et al. [17] conducted in vitro experiments using HeLa cells transfected with GJB3 and found that the expression of the junction protein supports the invasion of tumor cells into normal tissues, and this process does not require heterotypic gap junction coupling. As a member of the S100 protein family,Studies have shown that S100A16 overexpression was associated with varying tumor occurrence and progression rates [10, 11, 18]. High S100A16 expression in lung adenocarcinoma, ovarian cancer, cervical cancer and breast cancer were associated with a poor prognosis, whereas high expression in colorectal cancer and oral squamous cell carcinoma was associated with a favorable prognosis [8–11, 19, 20]. By analyzing the differences in S100A16 expression between tumor tissues and normal tissues in the TCGA datasets, we found that S100A16 expression was significantly increased in tumor tissue samples, and cervical cancer patients with high S100A16 expression had more rapid disease progression and shorter survival time. These results implicate S100A16 as potential therapeutic target in cervical cancer. However, the exact mechanisms by which it affects tumor progression remain unclear and a topic for further investigation.

The tumor microenvironment has a significant impact on cancer diagnosis, survival outcome, and sensitivity to clinical treatment. Among them, the immune system establishes the microenvironment adaptive to the cancer cells through modulating various cytokines and chemokines, thereby promoting or repressing tumor growth [21]. Tumor cells leverage the autogenous regulation of the immune system to build an immunosuppressive network in the tumor microenvironment. Cells regulating the immunosuppressive network include regulatory T cells (Tregs), dendritic cells (DCs), and natural killer cells (NKs) [22]. Because of the interaction between tumor cells and immune cells, large quantities of regulatory cells and inhibitory factors that are detrimental to immune response and favorable to tumor growth, migrate to the vicinity of tumor cells [23]. These immune effectors and suppressors are closely related to cancer development. On the other hand, the tumor microenvironment may also influence the metabolic reprogramming of tumors, maintaining immune cell function and modulating the immune system through metabolic competition and symbiosis [24]. Through the relationship between hub genes and tumor immune infiltration from the TCGA datasets, this study investigated the potential molecular mechanisms of hub genes and cervical cancer progression. We discovered that S100A16 was significantly positively correlated with resting mast cells, dendritic cells, and activated t cells, and significantly negatively correlated with naïve B cells and resting CD4 memory T cells. Inside tumors [25], MCs interact with the infiltrated immune cells, cancer cells, and extracellular matrix through direct cell-to-cell interactions or release of mediators capable of reshaping the tumor microenvironment. By releasing the classical or non-classical proangiogenic factors, MCs actively promote angiogenesis and induce neovascularization. In addition, MCs support tumor invasion by releasing extensive matrix metalloproteinases (MMPs) [18]. Furthermore, tumor cells induced the differentiation of neighboring dendritic precursors to the Gr-1( +) conventional dendritic cell subpopulation and binding with cytotoxic T-lymphocyte-associated antigen 4 (CTLA-4), suppressing their proliferation and promoting the immune escape of cancer cells. Thus, inhibiting Gr-1( +) and CTLA-4 can improve tumor immune response [26]. In the present study, the positive correlation of dendritic cells and S100A16 confirmed previous findings. T cells are produced by activated monocytes/macrophages in lymph nodes. Through the expression of T cell receptor (TCR) α/β, CD4^+^ or CD8^+^ T cells recognize tumor antigens and autoantigens and act on specific cancer cells, thereby exerting anti-tumor immunity. For instance, miR-18a acts by inhibiting proliferation and inducing cell death of CD4^+^ T cells [27]. Moreover, Tregs often accumulate in tumor tissues maintaining the immunosuppressive environment of the tumor and facilitating invasion and metastasis [28, 29].

Genomic instability is one of the hallmarks of cancer, characterized by an increased rate of changes in the cell genome, which promotes cancer progression and resistance to treatment. The most common form of genomic instability in cancer is chromosomal instability, driving uncontrolled cell proliferation and tumor development [30]. CC cells also exhibit abnormal chromosomes, which contain various gene rearrangements, including translocations, deletions, and gene amplifications. Given the importance of spindle-kinetochore interactions during cell division, any defects in mitosis are associated with chromosomal instability. The instability may be attributed to chromosomal segregation defects [31].

During tumor development, the enhancement of glycolysis in cells is mainly due to irreversible damage to oxidative phosphorylation function. The mechanisms leading to oxidative phosphorylation pathway damage and the primary energy metabolic pathways (glycolysis and oxidative phosphorylation) relied upon differ among different types of tumor cells. However, almost all tumor cells exhibit varying degrees of impairment in mitochondrial oxidative phosphorylation function. The specific mechanisms still require further in-depth research.

High expression of the S100A16 protein was correlated to disease progression and prognosis of cervical cancer, and thus we investigated the hub genes and sensitivities to common chemotherapeutic drugs [32]. Our study results suggest that cervical cancer patients with high S100A16 expression were highly sensitive to drugs, such as gemcitabine and axitinib. Treating SiHa cells using an agonistic anti-CD40 monoclonal antibody or gemcitabine alone could not inhibit the proliferation of SiHa cells in vitro, whereas the activation of CD40 on SiHa cells enhanced their sensitivity to gemcitabine [32]. Preliminary research showed that when used concomitantly with cisplatin, gemcitabine may be helpful in the treatment of recurrent or advanced cervical cancer [33].

The biological functions of S100A16 in tumors remain obscure. Studies have shown that S100A16 activates the AKT signaling pathway in prostate cancer to promote cell invasion, metastasis, and proliferation [18]. Additionally, the activation of the AKT cellular pathway promotes cell survival and inhibits apoptosis [34, 35]. S100A16 plays a crucial role in cisplatin resistance during chemotherapy for the treatment of lung cancer. Zhou et al*.* reported that S100A16 promotes epithelial to mesenchymal transition (EMT) through the Notch pathway in breast cancer [36], while EMT enhanced the invasion and metastasis of epithelial cells and was associated with resistance to chemotherapy in a variety of tumors [37, 38]. On the contrary, in oral squamous carcinoma, the prognosis was better for patients with high S100A16 expression [10] suggesting that S100A16 had different buffering or inhibitory functions in adenocarcinoma and squamous carcinoma in an expression-dependent manner. A study by Li et al*.* showed that S100A16 enhanced the expression of TWIST1 by activating the STAT3 signaling pathway, which subsequently promoted the EMT and invasiveness of pancreatic cancer cells [39]. In addition, inhibition of S100A16 expression could slow the metastasis of pancreatic cancer cells. Ou et al*.* showed that S100A16 could repress the proliferation, migration, and invasion of colorectal cancer cells through the JNK/p38 MAPK pathway [40]. A study by Zhang et al*.* showed that S100A16 [41] promoted the proliferation, migration, and tumor angiogenesis of cervical cancer HeLa cells via regulating the PI3K/PKB signal transduction pathway, while the PI3K/PKB signaling pathway was closely related to cancer proliferation, invasion, differentiation, and drug resistance [42, 43]. Zhu et al. showed that S100A16 could promote the proliferation of prostate cancer cells through the AKT and ERK signaling pathways [18]. The results of this study showed that high S100A16 expression significantly enriched the p53 and apoptosis pathways. In addition, research has shown that CIZAR induced apoptosis not only by reinstating the p53/Rb-dependent pathway in HPV-positive cells, but also by activating the p53/Rb-independent pathway and mitochondrial death signaling pathway in cervical carcinoma cells, which was unrelated to HPV infection [44]. SOX14 overexpression in cervical carcinoma triggered the accumulation of p53, indicating that potential interactions exist between the SOX14 and p53 signaling pathways [45]. 

## Conclusions

In summary, S100A16 mRNA and protein were abnormally upregulated in cervical cancer, and their overexpression indicated a poor prognosis and malignant tumor progression in cervical cancer patients. These results preliminarily present an opportunity for improvement in the diagnosis and treatment of cervical cancer, by revealing S100A16 as a new target for cervical cancer therapy and uncovering its apparent role in cellular susceptibility to an array of current chemotherapeutic drugs. Global results of this study also implicated S100A16 as an up-regulator of multiple tumor growth progression processes, highlighting the potential importance of continued investigation and further studies into the pathogenic mechanisms of S100A16 in cervical cancer cells and its relationship to immune response and key cellular pathways. 

## Data Availability

The initial data used to support the findings of this study are available from the corresponding author upon request. Publicly available datasets were analyzed in this study. This data can be found here: TCGA-CESC (https://portal.gdc.cancer.gov/). GSE44001 (https://www.ncbi.nlm.nih.gov/geo/query/acc.cgi?acc=GSE44001).
